# Seizure-induced overexpression of NPY induces epileptic tolerance in a mouse model of spontaneous recurrent seizures

**DOI:** 10.3389/fnmol.2022.974784

**Published:** 2022-10-13

**Authors:** Meinrad Drexel, Günther Sperk

**Affiliations:** Department of Pharmacology, Medical University Innsbruck, Innsbruck, Austria

**Keywords:** neuropeptide Y, somatostatin, CB1 receptor, subiculum, hippocampus, ischemic tolerance, parvalbumin, O-LM cells

## Abstract

Epileptic seizures result in pronounced over-expression of neuropeptide Y (NPY). *In vivo* and *in vitro* studies revealed that NPY exerts potent anticonvulsive actions through presynaptic Y2 receptors by suppressing glutamate release from principal neurons. We now investigated whether seizure-induced over-expression of NPY contributes to epileptic tolerance induced by preceding seizures. We used a previously established animal model based on selective inhibition of GABA release from parvalbumin (PV)-containing interneurons in the subiculum in mice. The animals present spontaneous recurrent seizures (SRS) and clusters of interictal spikes (IS). The frequency of SRS declined after five to six weeks, indicating development of seizure tolerance. In interneurons of the subiculum and sector CA1, SRS induced over-expression of NPY that persisted there for a prolonged time despite of a later decrease in SRS frequency. In contrast to NPY, somatostatin was not overexpressed in the respective axon terminals. Contrary to interneurons, NPY was only transiently expressed in mossy fibers. To demonstrate a protective function of endogenous, over-expressed NPY, we injected the selective NPY-Y2 receptor antagonist JNJ 5207787 simultaneously challenging the mice by a low dose of pentylenetetrazol (PTZ, 30 or 40 mg/kg, i.p.). In control mice, neither PTZ nor PTZ plus JNJ 5207787 induced convulsions. In mice with silenced GABA/PV neurons, PTZ alone only modestly enhanced EEG activity. When we injected JNJ 5207787 together with PTZ (either dose) the number of seizures, however, became significantly increased. In addition, in the epileptic mice CB1 receptor immunoreactivity was reduced in terminal areas of basket cells pointing to reduced presynaptic inhibition of GABA release from these neurons. Our experiments demonstrate that SRS result in overexpression of NPY in hippocampal interneurons. NPY overexpression persists for several weeks and may be related to later decreasing SRS frequency. Injection of the Y2 receptor antagonist JNJ 5207787 prevents this protective action of NPY only when release of the peptide is triggered by injection of PTZ and induces pronounced convulsions. Thus, over-expressed NPY released “on demand” by seizures may help terminating acute seizures and may prevent from recurrent epileptic activity.

## Introduction

Ischemic or epileptic tolerance are mechanisms contributing to termination of seizures and protect from exacerbation of epilepsy (Gidday, 2006; Simon et al., 2007). Epileptic tolerance may be caused by reorganization of neuronal circuitries or expression of endogenous anticonvulsive compounds. For example, erythropoietin (Sakanaka et al., 1998), adenosine (Schubert and Kreutzberg, 1993), neurosteroids (Biagini et al., 2010; Lawrence et al., 2010) and heat shock proteins (HSP) over-expressed after ischemia or epileptic seizures protect from subsequent seizures (Chang et al., 2014). Seizures also significantly activate GABA-ergic interneurons and induce augmented expression of the GABA synthesizing enzyme glutamate decarboxylase 67 (GAD-1) and of neuropeptides contained in GABA neurons (Houser et al., 1986; Marksteiner and Sperk, 1988; Feldblum et al., 1990; Sperk et al., 1992).

Particularly the expression of neuropeptide Y (NPY) is strongly enhanced in animal models of epilepsy and human temporal lobe epilepsy (TLE). Kainic acid (KA)-induced seizures or kindling induce a widespread and lasting over-expression of NPY in the hippocampus and in cortical areas (Marksteiner and Sperk, 1988; Schwarzer et al., 1996; Drexel et al., 2012). In the dentate gyrus of the hippocampus, its expression is transient in granule cells but lasting in interneurons (Gruber et al., 1994) and associated with transient upregulation of Y2 but not Y1 receptors in mossy fibers (Röder et al., 1996; Kofler et al., 1997; Schwarzer et al., 1998; Furtinger et al., 2001). Seizures markedly enhance expression of the peptide also in interneurons of the hippocampus proper and in the subiculum (Vezzani and Sperk, 2004; Drexel et al., 2012). NPY and Y2 receptor agonists, like NPY (3-36) as well as viral vectors expressing NPY are strongly anticonvulsive when applied *in vitro* or in living animals (Woldbye et al., 1996, 2010; Richichi et al., 2004; El Bahh et al., 2005; Ledri et al., 2015). This activity is mediated by potent inhibition of glutamate release through NPY-Y2 receptors located presynaptically on glutamate neurons (Colmers et al., 1991; Greber et al., 1994). There is also evidence that endogenous NPY may be involved in seizure control. Knock out of NPY or of Y2 receptors makes mice more susceptible to epileptic seizures (Baraban et al., 1997; El Bahh et al., 2005) and, in reverse, transgenic rats over-expressing NPY are less susceptible to convulsant drugs or kindling (Vezzani et al., 2002). A protective role of endogenous, over-expressed NPY has been documented in seizures induced by elevated temperatures in 10–11 days old rat pups (Dubé et al., 2005). Whereas in naïve rat pups seizures were induced by rising the surrounding temperature to 41°C, the threshold for initiating seizures increased by one and two°C when hyperthermia was repeated after 4 and 8 h, respectively, indicating that the rats became somewhat resistant to the rise in temperature. Concomitantly with the first exposure to hyperthermia, an increase of NPY mRNA expression was observed in interneurons of the dentate hilus, and pretreatment with the Y2 receptor antagonist BIIE0246 antagonized the NPY mediated epileptic tolerance (Dubé et al., 2005).

We now investigated whether endogenous NPY released after its over-expression during spontaneous recurrent seizures (SRS) may protect from subsequent seizures and thereby may induce epileptic tolerance. We used an animal model of increased seizure susceptibility after silencing parvalbumin (PV) interneurons in the subiculum and show increased expression of NPY and precipitation of acute seizures by antagonizing NPY-Y2 receptors. The experiments indicate that NPY over-expressed by SRS contributes to epileptic tolerance by activating Y2 receptors. Analogous to our previous studies (Drexel et al., 2012, 2017) we focused also in our present study on the ventral subiculum/hippocampus. We aimed to target the subiculum as the primary output region of the hippocampus, which is anatomically closely related to the ventral hippocampus in rodents and better accessible for the required vector injections. In addition is the ventral hippocampus a primary epileptogenic zone in rat models with high relevance for human TLE (Buckmaster et al., 2022).

Besides NPY a variety of other parameters of GABA-ergic neurons, e.g., glutamate decarboxylase (GAD1 and 2), somatostatin (SOM) or cholecystokinin (CCK-8) are dynamically altered in animal models of epilepsy (Schwarzer et al., 1996). A loss of dynorphin contained in mossy fibers is associated with the seizure presentation which can be overcome by substitution of the peptide by kappa opioid agonists (Solbrig et al., 2006a,b). Another possible mechanism for an endogenous adaptive and anticonvulsive mechanism may be down-regulation of the cannabinoid receptor CB1. Endocannabinoids are released by CCK-8 containing basket cells and can retrogradely inhibit GABA release through presynaptic CB1 receptors and thereby have pro-convulsive effect (Katona et al., 1999). After KA-induced epilepsy that is associated with a loss of subicular PV neurons, we observed sprouting of SOM neurons in the outer molecular layer of the subiculum (Drexel et al., 2017), which may serve an endogenous anticonvulsive function (Vezzani and Hoyer, 1999). Thus, in the search of possible alternative mechanisms mediating epileptic tolerance we investigated VGAT-, SOM-IR and mRNA expression, dynorphin-IR and CB1-IR in the hippocampus of mice injected with AAV-*TeLC* or AAV-*GFP*.

## Materials and methods

### Mice

Animal experiments were conducted according to national guidelines and European Community laws and were approved by the Committee for Animal Protection of the Austrian Ministry of Science. PV-cre transgenic mice [Pvalb^TM1(cre)Arbr^; Jackson Laboratories, Farmington, CT, United States, obtained through Charles River, Sulzfeld, Germany], expressing Cre-recombinase under the PV promoter, were maintained on a C57BL/6N background. All mice were housed in groups of 3–5 in single ventilated cages under standard laboratory conditions (12/12 h light/dark cycle, light turns on at 06:30 AM) and had free access to food and water. For the experiments, 10–14 weeks old heterozygous male mice [Pvalb^TM1(cre)Arbr±^] were used.

### Vectors

Adenovirus associated virus vector (AAV1/2) containing either the gene for tetanus toxin light chain (TeLC) fused with a green fluorescence protein (GFP) tag or GFP alone, with the reading frames inverted in a flip-excision (FLEX) cassette (AAV-*TeLC* and AAV-*GFP*, respectively) and a cytomegalovirus (CMV) immediate early enhancer/chicken β-actin promoter were prepared as described (Murray et al., 2011; Drexel et al., 2017).

### Surgery and vector injection

Anesthesia and stereotaxic surgeries were performed as described in detail previously (Drexel et al., 2017). One hour prior to surgery, the mice were injected with the analgesic drug carprofen (5 mg/kg, s.c.; Rimadyl, Pfizer, United States). They were then anesthetized with 150 mg/kg i.p. ketamine (Ketasol, stock solution 50 mg/ml, Ogris Pharma Vertriebs-GmbH, Wels, Austria) and maintained anesthetized under 1–3% sevoflurane (Sevorane, Abbott, Vienna, Austria) applied through a veterinary anesthesia mask with oxygen (400 ml/min) as a carrier gas. For surgery, the anesthetized mice were placed into a stereotaxic frame (David-Kopf Instruments, Tujunga, CA, United States) and the skin above the skull was opened. A telemetric EEG transmitter (TA10EA-F20, Data Sciences International, Arden Hills, MN, United States) was inserted into a subcutaneous pocket at the abdominal wall and the electrode wires were pulled through a subcutaneous channel from the pocket to the skull, and the pocket was sutured. Bilateral holes were drilled for AAV-vector injection and insertion of a depth-electrode (coordinates from bregma in mm: posterior, 3.8; lateral, 3.5) and for the epidural reference electrode (posterior, 2.0; lateral, 1.6).

We injected the AAV vectors (1.5 μl), AAV-*TeLC* (titer: 2.4 × 10^10^/ml) or AAV-*GFP* (1.4 × 10^11^/ml for pAM-FLEX-*GFP*, for controls) unilaterally at the above coordinates into the left ventral CA1/subiculum (3.5 mm deep). For telemetric EEG recordings, we implanted a tungsten depth electrode (Cat. no 577100; Science Products GmbH, Hofheim, Germany) into the left ventral subiculum (3.0 mm deep) and attached, as reference electrode, a stainless-steel screw (M1*2, Hummer und Rieß GmbH, Nürnberg, Germany) epidurally to the skull (posterior, 2.0, right, 1.6). Electrodes were fixed to the skull with dental cement (Paladur, Heraeus Kulzer, Henry Schein, Innsbruck, Austria). After surgery and during EEG recording, the mice were single housed.

C57BL/6N wild-type mice (Charles River, Sulzfeld, Germany) or PV-cre transgenic mice injected with AAV-*GFP* into CA1/subiculum were used as controls.

### EEG recordings and video monitoring

EEGs were recorded continuously using a telemetry system (Dataquest A.R.T. Gold 4.33 Acquisition, Data Sciences International, Arden Hills, MN, United States). For recording motor seizures in addition to EEG seizures, continuous video recordings were performed with Axis 221 network cameras (Axis communications AB, Lund, Sweden) and, during darkness, under infrared illumination (Conrad Electronic GmbH, Wels, Austria). EEGs were recorded at a sampling rate of 1,000 Hz without *a priori* filter cut-off and saved on external hard disk drives.

EEG traces of local field potentials were visually inspected by two independent observers using the Dataquest A.R.T. Gold 4.33 Analysis software. We defined seizures as EEG segments with continuous high frequency activity with an amplitude of at least twice the baseline amplitude, a duration of at least 10 s, and the presence of a post-ictal depression (EEG-signal below baseline amplitude). Clusters of interictal spikes (IS) were defined as a series of at least 5 high amplitude (at least 2 times baseline) discharges not more than 60 s apart.

After terminating EEG recordings 42 (Experiment 1) or 102 days (Experiment 2) after vector injection, the mice were deeply anesthetized with thiopental (150 mg/kg; Sandoz, Austria) and perfused *via* the left ventricle of the heart with 25 ml 50 mM phosphate-buffered saline (PBS, room temperature) followed by 100 ml ice-cold 4% paraformaldehyde (PFA). The brains were then removed from the skulls, post-fixed in 4% PFA/PBS (cold room, 90 min) and then transferred to 20% sucrose/PBS (cold room, over-night). The brains were then snap-frozen in –70°C isopentane (Merck, Darmstadt, Germany, 3 min) and kept in sealed vials at –70°C until they were cut in a cryostat-microtome (Carl Zeiss AG, Vienna, Austria). Microtome sections (40 μm) were maintained in PBS/0.01% sodium azide at cold room temperature (4°C) until they were processed.

### Evaluation of motor seizures

All seizures observed by EEG were investigated by video recordings for their behavioral correlate. Seizure rating was done in accordance with that used for KA-injected rats (Sperk et al., 1983). All EEG seizures corresponded to limbic motor seizures (rating 3–4; data not shown).

### Experimental approach

The goal of the experiments was to induce spontaneous seizures by selective, locally restricted silencing of PV containing interneurons by unilateral, local injection of AAV-*TeLC* into the ventral subiculum/CA1 area. We investigated two primary parameters in these mice: 1. Temporal patterns of SRS and of IS clusters, and 2. Expression of NPY-immunoreactivity (IR) in interneurons of the hippocampus and in mossy fibers of the dentate gyrus.

We performed three principal experiments (focused on NPY expression in relation to SRS) and one experiment investigating the role of the Y2 receptor antagonist JNJ 5207787.

#### Experiment 1

**FIGURE 1:**
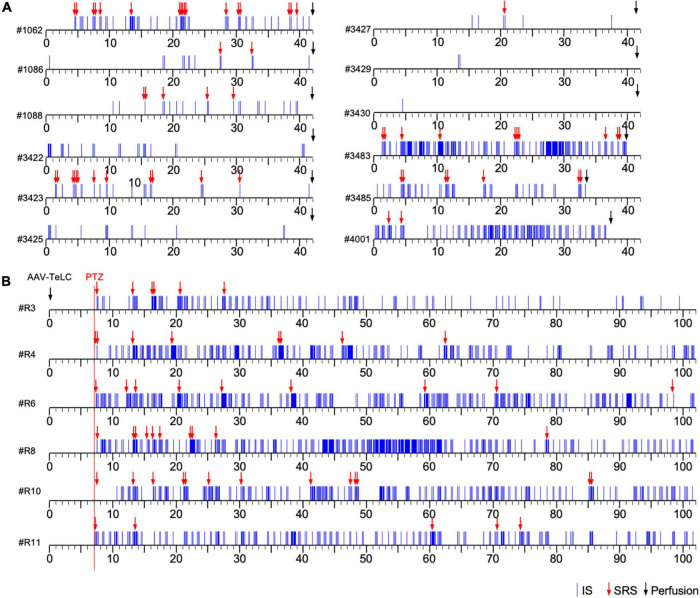
Development of clusters of interictal spikes (IS) and spontaneous recurrent seizures (SRS) in PV-cre mice after unilateral injection of AAV-*TeLC* into the ventral subiculum/area CA1. **(A)** Events of IS clusters (blue lines) and of SRS (red arrows) are listed for twelve representative mice; the respective identification code is shown left to the time scale. Panels **(A,B)** show the results of Experiments 1 and 2, respectively (see section “Experimental approach”). In contrast to Experiment 1 **(A)**, in Experiment 2 **(B)** EEG recordings were started at day seven after vector injection (red vertical line). About 30 min later, mice were injected with a low dose of PTZ (40 mg/kg; red arrows indicate acute PTZ-induced seizures). Besides acute seizures, PTZ induced development of SRS and IS clusters in all AAV-*TeLC*-injected mice but not in AAV-*GFP*-injected mice (not shown). In Experiment 1, mice were sacrificed after 42 days (except three mice that lost their electrodes earlier; **(A)**. In Experiment 2, mice were monitored for 102 days **(B)**. Both groups of mice were sacrificed immediately after terminating EEG recordings and their brains were processed for NPY-IR. Five AAV-*GFP*-injected mice were included as controls in each experiment. They developed no SRS or IS clusters (not shown).

We injected 22 mice with the AAV-*TeLC* vector into the ventral subiculum/CA1, monitored EEG and behavioral activity for 42 days (experimental details see below), sacrificed the mice and processed their brains for NPY-IR. Three mice lost their electrodes at earlier intervals and were therefore sacrificed at these points (Figure 1A shows 12 representative AAV-*TeLC* mice). Five mice were injected with AAV-*GFP* as controls and treated in the same way. They did not develop SRS or IS clusters. The numbers of animals are based on our previous experiments (Drexel et al., 2017) taking in account that about one third of mice will not show SRS.

#### Experiment 2

In the first experiment, we suspected that the frequency of SRS and IS clusters declined at later intervals after vector injection. For investigating this more closely, we performed a second experiment also silencing PV neurons in the ventral subiculum/CA1 by local AAV-*TeLC* injections. We injected six mice with AAV-*TeLC* and, according to our previous protocol (Drexel et al., 2017), triggered the development of SRS and IS clusters by a single low dose injection of PTZ (40 mg/kg, i.p.) 7 days after AAV-injection. All AAV-*TeLC*-injected mice developed SRS and IS (see Figure 1B). Five AAV-*GFP* injected mice were included as controls. None of them developed acute seizures upon PTZ application, SRS or IS. All mice were sacrificed after 102 days and their brains were processed for immunohistochemistry. The numbers of animals had been chosen according to our previous experiment using PTZ challenge after AAV-*TeLC* injection, resulting in 100% of mice developing SRS (Drexel et al., 2017).

#### Experiment 3

This experiment was performed for examining NPY mRNA by *in situ*-hybridization in the brains. Nine mice were injected with AAV-*TeLC* and with 40 mg/kg PTZ after 7 days. All mice developed SRS and IS clusters (not shown) and were sacrificed after 42 days. Eight mice were injected with AAV-*GFP* as controls; none of these mice developed acute seizures after PTZ injection, SRS or IS. The brains were snap frozen and processed for NPY mRNA *in situ* hybridization. The numbers of mice were chosen according to our previous experiments on NPY expression after KA-induced seizures (Drexel et al., 2012).

#### Experiment 4: Injection of the Y2 receptor antagonist JNJ 5207787

Mice were injected with the AAV-*TeLC* (*n* = 16) or with the AAV-*GFP* (*n* = 8) into sector CA1/subiculum. Recording electrodes were *placed* as described above and EEG recordings were started immediately after vector injection. One group of AAV-*TeLC*-injected mice received the non-peptidergic Y2 receptor antagonist JNJ 5207787 (30 mg/kg; Tocris, *N-*(1-Acetyl-2,3-dihydro-1H-indol-6-yl)-3-(3-cyano-phenyl)-*N-*[1-(2-cyclopentyl-ethyl)-piperidin-4yl]-acrylamide, Cat. No. 4018/10, Biotechne, Abingdon, United Kingdom) dissolved in 0.5 ml 2-hydroxy propyl-β-cyclodextrin (20%) in saline (80%) i.p. (*n* = 4) or 0.5 ml solvent alone i.p. (*n* = 3), and (after 30 min) 30 mg/kg i.p. PTZ in saline. A second group was injected with 0.5 ml solvent (*n* = 4) or with JNJ 5207787 (*n* = 5) followed by 40 mg/kg i.p. PTZ after 30 min. We injected also untreated controls (*n* = 8) and AAV-*GFP*-injected mice (*n* = 8) with JNJ 5207787 followed by 30 mg/kg PTZ and 40 mg/kg PTZ, respectively. EEG recordings were continued immediately after PTZ injections. JNJ 5207787 was injected 30 min prior to PTZ due to its much slower uptake to the brain compared to PTZ. JNJ 5207787 has been developed by Bonaventure et al. It is more than 100 times more active toward Y2 than Y1, Y4 or Y5 receptors *in vitro* and penetrates the brain at the dose applied (Bonaventure et al., 2004). The doses for PTZ have been optimized recently (Drexel et al., 2017).

### Immunohistochemistry

Immunohistochemistry for NPY was performed on free-floating, 40 μm thick horizontal 4% PFA-fixed sections (obtained in Experiment 1) using indirect peroxidase labeling as described before (Sperk et al., 2012; Wood et al., 2016). In brief, horizontal sections were incubated free-floating with 10% normal goat or horse serum (Biomedica, Vienna, Austria) in Tris–HCl buffered saline (TBS; 50 mM, pH = 7.2) containing 0.4% Triton X-100 (TBS-Triton) for 90 min and then incubated with a rabbit NPY antiserum raised toward the peptide coupled to ovalbumin (1: 1,000; (Marksteiner and Sperk, 1988) at room temperature for 16 h) and subsequently washed with TBS-Triton. The reaction product was visualized by incubation with a horseradish peroxidase (HRP)-coupled secondary antibody (1:250, goat anti-rabbit P0448; Dako, Vienna, Austria; 1:500; room temperature for 150 min). After washing with TBS, HRP bound to the secondary antibodies was reacted with 0.05% diaminobenzidine tetrahydrochloride dihydrate (DAB, Fluka, Sigma-Aldrich Handels GmbH, Vienna, Austria) and 0.005% H_2_O_2_ as substrate. Sections were then washed in TBS, mounted on gelatin-coated glass slides in 55% ethanol and allowed to dry overnight. After dehydration in ethanol and clearing in butyl-acetate, they were coverslipped using Eukitt mounting medium (Gröpl, Vienna, Austria).

Using sections obtained in Experiment 1, we furthermore studied possible changes in the expression of VGAT, SOM, dynorphin and CB1. Immunohistochemistry for SOM and dynorphin was performed as described previously using the same antibodies (Schwarzer et al., 1996; Pirker et al., 2001). VGAT-IR and CB1-IR were assessed by immunofluorescence using respective antibodies [VGAT (rabbit), donated by Prof. R. Edwards, University of California San Francisco, CA; 1:2,500 dilution (Sperk et al., 2003); CB1, CB1-Rb-Af380 (rabbit, RRID:AB_2571591, Hölzel Diagnostika Handels GmbH, Köln, Germany), dilution 1:1,000].

### *In situ* hybridization

NPY *in situ* hybridization was done in 20 μm thick horizontal microtome sections obtained from snap frozen sections. Tissue preparation and *in situ* hybridization was performed as described in detail previously (Drexel et al., 2012). Densitometry was done as described in detail before (Drexel et al., 2013). *In situ* hybridization for SOM and VGAT mRNAs was performed as described previously (Schwarzer et al., 1996; Sperk et al., 2003).

### Semi-quantitative analysis of NPY-IR in the molecular layer of the subiculum

Densitometry of NPY-IR was performed in microphotographs of 40 μm thick horizontal sections labeled by NPY immunohistochemistry. The sections were photographed at 10-fold magnification under consistent camera settings (including illumination times) with a black and white camera (Orca II, Hamamatsu Photonics, Hamamatsu City, Japan) attached to a Zeiss Axiophot microscope (Carl Zeiss GmbH, Jena, Germany). Two matched sections per mouse were used and values obtained from left and right hemispheres and from different sections were averaged.

Microphotographs (8 bit) were imported into NIH ImageJ (v. 1.53q^1^ ; NIH, Bethesda, MD, United States). To measure gray scale values of NPY-IR in mossy fibers, a line scan (width: 150 μm) through the mossy fibers in stratum lucidum of sector CA3a was performed. Gray scale values were converted to relative optical density (ROD)-values by the following formula: ROD = log [256/(255 - gray value)]. Section background-labeling was measured in the thalamus and subtracted from the values obtained in stratum lucidum. To measure NPY-IR in the molecular layer of the ventral subiculum, a line scan (width: 400 μm) was performed extending from the fissure between the dentate gyrus and the subiculum to the pyramidal cell layer of the subiculum. The resulting gray scale values were converted to ROD-values (see above), the curve was plotted, and the area under the curve above the molecular layer of the subiculum was calculated. The intensity of NPY-IR in the molecular layer of the subiculum was then given as arbitrary units (a.u., see graphs in Figures 4E, 5E).

### False color images

Microphotographs of NPY-immunolabeled sections were opened in NIH ImageJ and the image type was changed to 8 bit. “Spectrum” was selected as the lookup table and the colors of the lookup table were adjusted as follows: Black was selected for the bottom 4 rows of the lookup table and blue for the 5th row from the bottom. Brightness and contrast were optimized using identical values for all microphotographs.

### Statistical analyses

All statistical analyses were performed using GraphPad Prism statistical software (version 5.0f; GraphPad Software). The Fisher’s exact test was used for analysis to compare the number of SRS in mice 15–50 and 65–100 days after vector injection. Two-way repeated-measures ANOVA with Dunnett’s multiple-comparison *post hoc* test were used for comparing multiple groups differences in NPY-IR. A *p* value of < 0.05 was considered as statistically significant.

## Results

### Induction of spontaneous recurrent seizures (SRS) and interictal spike (IS) clusters by injection of AAV-*TeLC* into the subiculum/CA1

Unilateral injection of the AAV-vectors into the ventral subiculum/sector CA1 resulted in selective silencing of PV-containing basket and axo-axonic cells, as characterized in detail previously (Drexel et al., 2017). The ventral extension of the subiculum had been chosen as target for the vector injections because it is better accessible compared to the dorsal subiculum. Figures 1A,B show representative time courses of IS clusters and of SRS presented after injection of AAV-*TeLC* into the subiculum/CA1 (Experiments 1 and 2). As shown in Figure 1A (Experiment 1), most AAV-*TeLC*-injected mice experienced between one and 17 SRS and all mice exposed at least one IS cluster during the of 42 days period investigated. The first SRS was observed on day two after AAV-*TeLC* injection. IS clusters developed in all mice at considerably different frequencies; the first IS clusters were observed between days 1 and 14 after AAV-*TeLC*. None of the AAV-*GFP*-injected mice showed SRS or IS. The frequency of SRS appeared to decline gradually after around day 25.

**FIGURE 2:**
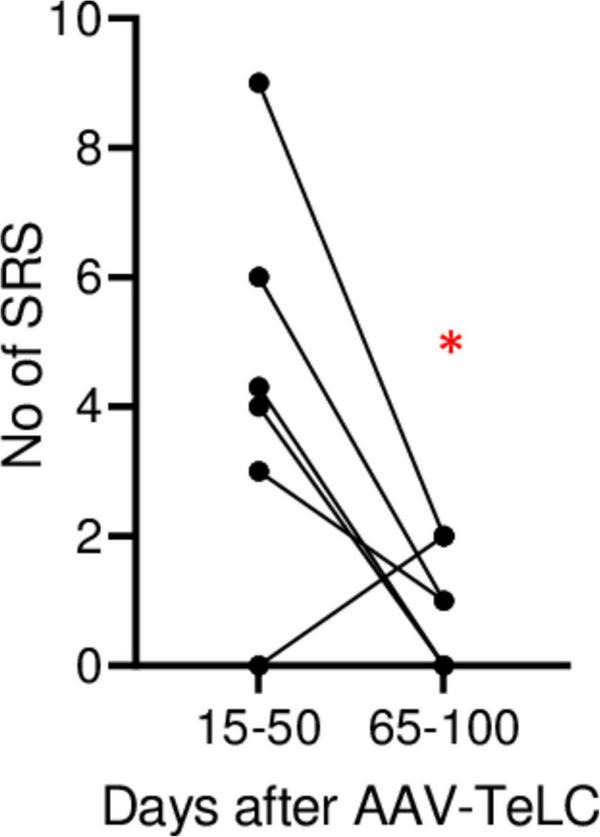
Decline in SRS frequency. The analysis was done for animals in Experiment 2 (monitored for 102 days). The graph shows the number of SRS for each mouse during days 15–50 and during days 65–100, respectively. The mean numbers of seizures were 4.3 ± 1.23 and 1.0 ± 0.37, respectively. At the 65–100 days interval, seizure frequency was significantly lower (*P* = 0.0452) in paired Student’s *t*-test (*). The data indicate development of induced epileptic tolerance.

In Experiment 2 (Figure 1B), we triggered SRS by injecting the convulsive GABA_A_ receptor antagonist PTZ 7 days after AAV-*TeLC* injection. The mice experienced between four to twelve SRS and numerous IS during the 102 days of EEG recording (Figure 1B). To determine whether the frequency of SRS declined at prolonged intervals after vector injection, we determined the mean seizure frequency at an early interval (15–50 days) after vector injection and compared it with that at a late interval (65–100 days; Figure 2). Between 15 and 50 days after AAV-*TeLC* injection, the mean number of seizures was 4.3 ± 1.23 and after 65–100 days (1.0 ± 0.37) it was significantly lower (*P* = 0.0452) indicating seizure-induced epileptic tolerance (see Figure 2).

### Differential increases of NPY expression in interneurons and in mossy fibers are related to SRS but not IS

**FIGURE 3:**
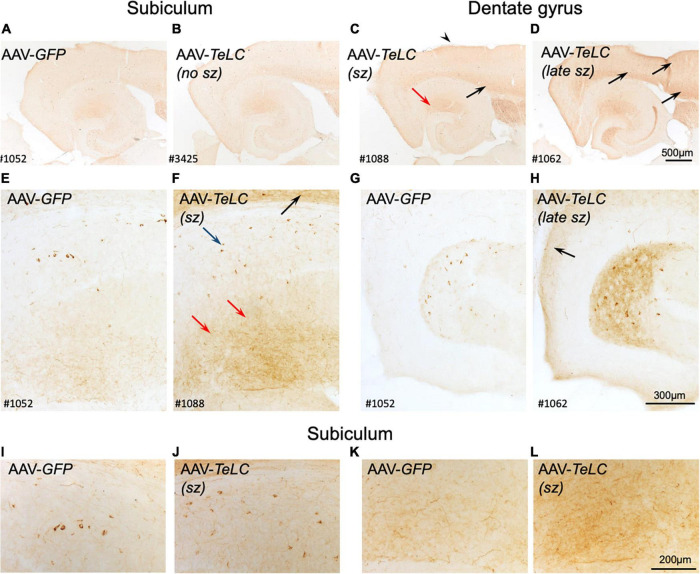
Expression of NPY-IR in the cortical areas, the subiculum and in the dentate gyrus 42 days after vector injection. Panels **(A,E,G)** show images from horizontal sections at the level of the of the ventral subiculum/hippocampus obtained from an AAV-*GFP*-injected mouse and show basal NPY expression. Panel **(B)** is taken from an AAV-*TeLC*-injected mouse without spontaneous seizures. In panel **(C)**, a mouse that had experienced 5 seizures between days 16 and 29 is shown (“sz”; see Figure 1A). Slightly increased expression of NPY-IR is seen in the outer molecular layer of the subiculum, in cortical layers [black arrows in panels **(C,D,F)**] and in hilar interneurons. Panels **(D,F,H)** derive from an AAV-*TeLC*-injected mouse that had experienced seven SRS during days 30–42 after vector injection (last 12 days before sacrificing, “late sz”). Note the pronounced increases in NPY-IR in cortical areas [arrows in panels **(D,F,H)**], in interneurons (blue arrow) and their fibers (red arrows) in the molecular layer of the subiculum **(D,F)**, and in interneurons and their fibers in the hilus of the dentate gyrus **(H)**; compare AAV-*GFP*-injected control **(G)**. Note that NPY-IR is lastingly up-regulated, both in interneurons and axon terminals of the subiculum [blue and red arrows in panel **(F)**] and of the dentate hilus **(H)**. Furthermore, panels **(I–L)** show larger magnification aspects of the subiculum highlighting the seizure-induced increased number of NPY-positive interneurons **(J)** and fibers **(L)** after AAV-*TeLC* injection compared to AAV-*GFP*-injected controls **(I,K)**. Numbers (#) in the left lower corners of the images refer to numbers of individual animals; the time course of their seizure spells and of IS clusters is shown in Figure 1A. sz, seizures between days 16 and 29 (seizure-free for last 13 days before sacrificing; sz +, strong seizures within 12 days before sacrificing.

**FIGURE 4:**
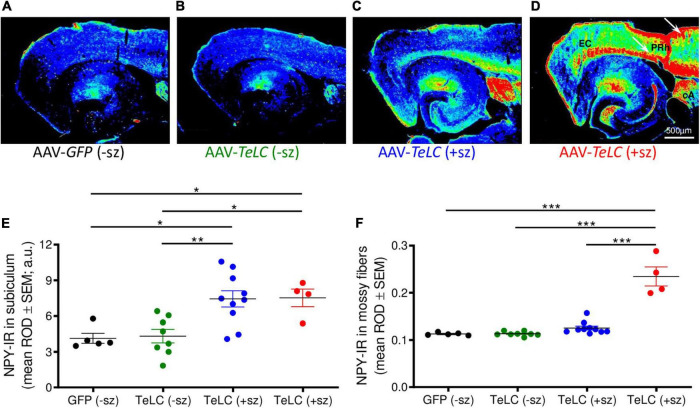
NPY overexpression in mice experiencing SRS but not in mice with clusters of IS. NPY-IR was assessed in horizontal brain slices at the level of the ventral subiculum/hippocampus in mice 42 days after injection with AAV-*GFP* or AAV-*TeLC*, respectively (Experiment 1). Panels **(A–D)** show false color images of NPY-IR representative for: **(A)** a control (AAV-*GFP*-injected, black); **(B)** representative mouse presenting only IS clusters after AAV-*TeLC* injection (green, mouse #3425); **(C)** a mouse that experienced SRS during days 10–30 (blue, mouse #1088); and **(D)** a mouse with seizures (3) after day 32 (red, mouse #1062). **(E,F)** Corresponding to the labeling of the images above, the first column in panels **(E,F)** shows data of control mice without SRS. Note the pronounced increase in NPY-IR in the subiculum, both in mice with seizures up to day 30 **(C)** and during 10 days before sacrificing **(D)**. Note also the strong increases in NPY-IR in cortical areas in epileptic mice [**(C)**, white arrows in panel **(D)**]. Black: AAV-*GFP*-injected controls (*n* = 5); green: AAV-*TeLC*-injected mice with IS only (*n* = 8); blue: mice that experienced SRS only in the first 32 days after AAV-*TeLC* injection (thus, were at least 10 days seizure-free before sacrificing) (*n* = 10); red: mice that experienced SRS within 5 days before sacrificing (*n* = 4). Note that the increase in NPY-IR persists in the subiculum of mice that experienced SRS only at earlier intervals [up to day 32 after vector injection; filled blue circles in panel **(E)**]. In contrast, increased expression of NPY-IR was observed in the dentate gyrus only in mice that showed SRS also within the last 5 days before sacrificing [filled red circles in panel **(F)**]. Letters in panel **(D)**: EC, entorhinal cortex, PRh, perirhinal cortex, cA, central amygdala. Statistical analysis was done by two-way repeated-measures ANOVA with Dunnett’s multiple-comparison *post hoc* test; ***p < 0.001; **p < 0.01; *p < 0.05.

**FIGURE 5:**
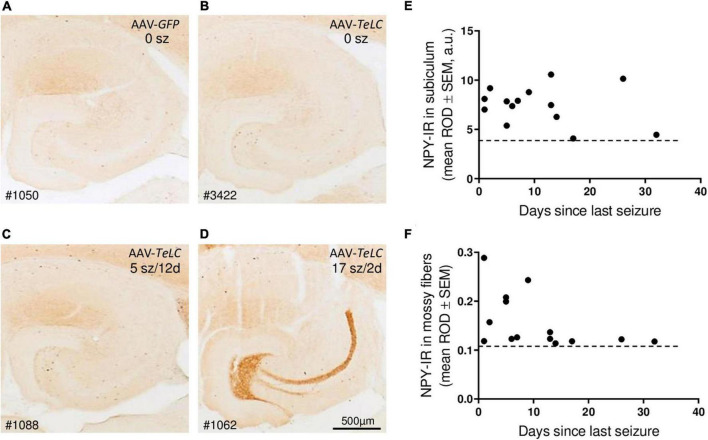
Seizure-induced over-expression of NPY-IR persists in interneurons of the subiculum but in mossy fibers is restricted to mice with frequent SRS close to sacrificing. NPY-IR in the ventral hippocampus at different intervals between last SRS and sacrificing. **(A)** AAV-*GFP*-injected control mouse (no seizures); **(B)** mouse that experienced IS only; **(C)** mouse that had expressed five SRS up to 12 days before sacrificing (last seizure on day 12; note increased NPY-IR in the inner molecular layer of the subiculum and in interneurons of the dentate gyrus) and **(D)** mouse that had 17 seizures in total, including three severe seizures two and three days before sacrificing (note the pronounced expression of NPY-IR in mossy fibers). **(E,F)** NPY-IR in relation of the time of last SRS to time of sacrificing in the subiculum **(E)** and in mossy fibers as determined in the stratum lucidum, the terminal area of mossy fibers **(F)**. Horizontal dotted lines indicate the background of NPY-IR. Note that in mossy fibers increased NPY-IR was detected only in mice that had experienced their last seizure 1–5 days prior to sacrificing **(F)**, whereas in interneurons of the subiculum the SRS-induced increase in NPY-IR persisted for up to 25 days **(E)**. Optic density is given in arbitrary units (a.u.).

We observed marked increases in NPY-IR throughout the brains of all mice with previous SRS and IS clusters (Figures 3C,D,F,H,J,L, 4C,D), but not in AAV-GFP-injected mice (Figure 3A) or in mice that had presented IS clusters alone (Figure 3B). The increases in NPY-IR were especially prominent in interneurons of the subiculum especially including their axon terminals in the outer molecular layer of the subiculum (Figures 3C,D,F,L). Increased NPY-IR was also observed in the cerebral cortex (Figures 3C,D, 4C,D, 5C) and in interneurons of the dentate gyrus (Figures 3H, 4C). Expression of NPY-IR in interneurons (of the subiculum/sector CA1 and of the dentate gyrus) was high, independently of the interval between the last seizure and sacrificing (Figures 5C,E); in contrast, in mossy fibers NPY-IR increased only in mice that experienced their last seizure during the last 5 days before sacrificing (Figures 5D,F; compare Figures 5E,F). In mice with longer intervals (7–15 days after last seizure) between the last seizure and sacrificing, NPY-IR had faded away in mossy fibers during the succeeding seizure-free interval (Figures 3C, 5C,F).

Thus, in the molecular layer of the subiculum (representing axon terminals of interneurons; see Figures 3F,L), NPY-IR was increased in mice that had experienced their last seizure already 10–21 days before sacrificing (179.6 ± 16.50%; *P* < 0.05 vs. controls) as well as in mice that had their last seizure within the last 5 days before sacrificing (181.6 ± 17.87%; *P* < 0.05 vs. controls) (Figure 4E). In contrast, in the dentate gyrus, NPY-IR was increased (207.5 ± 17.91%; *P* < 0.001 vs. controls) only in the four mice that experienced their last seizure within the last 5 days before sacrificing (Figure 4F) but not in the ones that experienced their last seizure more than 10 days before sacrificing (Figure 4E).

**FIGURE 6:**
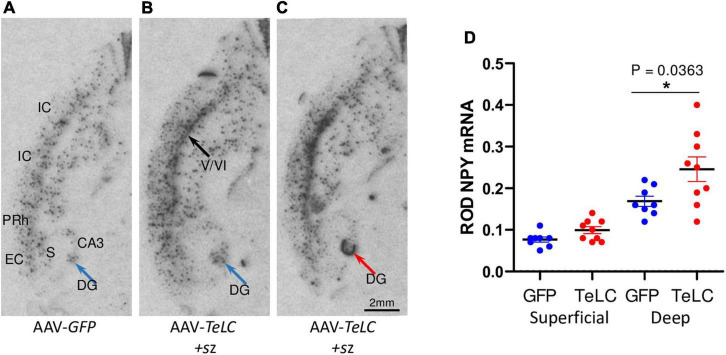
NPY mRNA expression in the deep layers of the cerebral cortex. Mice injected with AAV-*GFP* or AAV-*TeLC* were sacrificed after 6 weeks and brains were subjected to *in situ* hybridization. The images were obtained in horizontal sections showing the ventral hippocampus and adjacent limbic cortex areas (insular, perirhinal, entorhinal cortices). **(A)** AAV-*GFP*-injected control; **(B)** AAV-*TeLC*-injected mouse that exposed five SRS between days 6–25; **(C)** AAV-*TeLC*-injected mouse that exposed four SRS on days 3 and 2 before sacrificing (day 42). Black arrow in panel **(B)** denotes NPY mRNA expression in the deep cortical layers; blue arrow in panel **(B)** points to interneurons in the dentate hilus expressing NPY mRNA; red arrow in panel **(C)** denotes massive *de novo* NPY mRNA expression in the granule cell layer. **(D)** Quantification of NPY mRNA in the deep and superficial cortical layers 6 weeks after AAV-*GFP* and AAV-*TeLC* injection. Data are shown as mean ± SEM. Statistics (Student’s *t*-test) was performed using the GraphPad Prism program. In interneurons of the deep layers (V/VI) but not in the superficial layers (layers II/III) of the insular, perirhinal and entorhinal cortices, NPY mRNA was significantly increased (*P* = 0.0363, *). Abbreviations: DG, dentate gyrus (hilus); CA3, hippocampal subfield CA3; S, subiculum; IC, insular cortex; PRh, perirhinal cortex; EC, entorhinal cortex.

Like in axon terminals of subicular interneurons, also in interneurons of the dentate hilus (Figures 3G,H, 6B) and in the cerebral cortex (insular, perirhinal, entorhinal cortices; see Figure 6) the increases in NPY-IR appeared to be independent from the duration of the seizure-free interval before sacrificing. Thus, the persisting increase in NPY-IR in interneurons (notably of the subiculum), but not its transient increase in mossy fibers, may be causatively related to the subsequent decline in seizure frequency (epileptic tolerance).

### Injection of the NPY-Y2 receptor antagonist JNJ 5207787 facilitates PTZ-induced seizures

**FIGURE 7:**
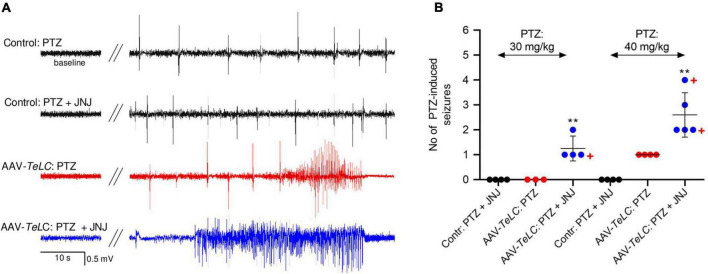
The Y2 receptor antagonist JNJ 5207787 potentiates PTZ-induced seizures in AAV-*TeLC*-injected epileptic mice. **(A)** EEG traces after injection of 40 mg/kg PTZ i.p. or injection of the Y2 receptor antagonist JNJ 5207787 (20 mg/kg, i.p.) followed by PTZ (40 mg/kg, i.p.). Note that neither the low dose of PTZ nor PTZ + JNJ 5207787 induced seizures in naïve controls (black traces) nor in AAV-*GFP*-injected mice (not shown). PTZ induced significant convulsions in AAV-*TeLC*-injected mice (red trace) that were markedly augmented by prior 20 mg/kg JNJ 5207787 injection (blue trace). **(B)** Numbers of PTZ-induced seizures with and without JNJ 5207787 injection. Neither naïve controls (black circles) nor AAV-*GFP*-injected mice (not shown; together *n* = 8) presented epileptic spells after 30 or after 40 mg/kg PTZ without JNJ 5207787 (*n* = 3 and 4, respectively; black filled circles). Mice previously injected with AAV-*TeLC* responded with no seizure (0/3) after 30 mg/kg PTZ; after 40 mg/kg PTZ all four mice responded modestly with 1 seizure (red filled circles). At both doses of PTZ, the number of seizures markedly increased when injected together with JNJ 5207787 (blue filled circles). Neither control mice (*n* = 4) nor AAV-*GFP*-injected mice (*n* = 3) showed changes in the EEG after JNJ 5207787 injection (not shown). +, indicate mice that died in seizures. Statistics (Student’s *t*-test): **p < 0.01, respective AAV-*TeLC* and PTZ-injected with vs. without prior injection of JNJ 5207787. The important message of this experiment is that the Y2 antagonist is inactive in naïve mice and in mice without acute seizures. However, in mice with seizures, it augments the epileptic activity presumably by inhibiting the protective action of NPY (released primarily during seizures) on Y2 receptors.

All mice were injected with PTZ (40 mg/kg, i. p.) 7 days after AAV-*TeLC* injection; as shown previously, this treatment resulted in SRS and NPY over-expression in 100% (Drexel et al., 2017). To demonstrate that over-expressed NPY serves an endogenous protective role, we injected 7 days later the Y2 receptor antagonist JNJ 5207787 (Figure 7). Neither controls nor AAV-*TeLC*-injected mice disclosed EEG changes after JNJ 5207787 alone (not shown). No seizures were observed in untreated controls or AAV-*GFP*-injected mice after injection of PTZ (30 or with 40 mg/kg) or PTZ plus JNJ 5207787 (black traces in Figure 7A and black filled circles in Figure 7B). Treating AAV-*TeLC*-injected mice with PTZ (30 mg/kg) alone revealed IS activity but no convulsions (EEG-trace not shown) and, after 40 mg/kg PTZ, in average one convulsion (Figures 7A,B; red traces and red filled circles, respectively). In mice injected with the Y2 receptor antagonist together with PTZ, we observed significant increases in EEG activity, including IS clusters and increased numbers of convulsions after 30 mg/kg PTZ + JNJ 5207787 (1.3 ± 0.25 convulsions vs. zero) and after 40 mg/kg PTZ + JNJ 5207787 (increase from 1.0 ± 0.00 to 2.6 ± 0.40 convulsions); one and two mice died in seizures after 30 and 40 mg/kg PTZ plus JNJ 5207787, respectively [Figure 7A blue trace (40 mg/kg PTZ); Figure 7B blue filled circles].

### VGAT, SOM, dynorphin and CB1 expression after SRS induced by AAV-*TeLC* injections

#### Dynorphin-IR

Dynorphin is highly expressed in mossy fibers and has been considered to act anticonvulsive through kappa receptors (Solbrig et al., 2006b; Loacker et al., 2007). As shown in Supplementary Image 1, we observed no change in dynorphin-IR in mossy fiber terminals in the stratum lucidum and in the dentate hilus of mice that had experienced series of SRS (see Figure 1). Interestingly, the mice revealed no sprouting of mossy fibers to the inner molecular layer of the dentate gyrus in spite of SRS, confirming that the mice had no loss in hilar interneurons.

#### VGAT-IR

The vesicular GABA transporter VGAT is a valid marker for GABA-ergic axon terminals. We investigated VGAT-IR in the hippocampus/subiculum for obtaining a possible indication for sprouting of GABA neurons. We observed, however, no change in the density or distribution of VGAT-IR 42 days after AAV-TeLC injection into the subiculum (Supplementary Image 2).

#### SOM mRNA and IR

**FIGURE 8:**
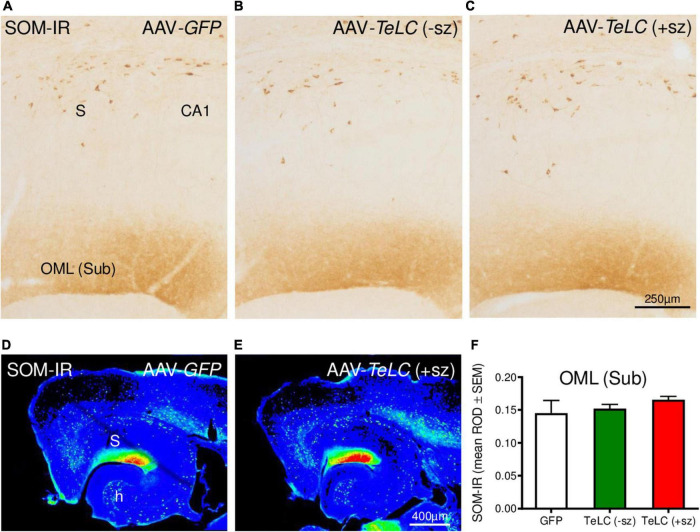
SOM-IR after AAV-*TeLC* injection. SOM-IR in sector CA1 and subiculum of mice injected with AAV-*GFP* **(A)** or AAV-*TeLC* that were seizure-free **(B)** or had experienced SRS **(C)**. Note that neither the number of SOM-positive interneurons in the pyramidal layers of the subiculum (S) and sector CA1 nor the density of SOM-positive axon terminals in the outer molecular layer (OML) of the subiculum was altered. Panels **(D,E)** show false color images of panels **(A,C)**, respectively. **(F)** Mean ROD values of the molecular layer were not altered by previous SRS. +sz, mouse with previous seizures; -sz, mouse was seizure-free.

We previously obtained indication for sprouting of SOM neurons in the molecular layer of the subiculum after KA-induced seizures in the rat (Drexel et al., 2012). Since this may represent an endogenous anticonvulsive mechanism, we investigated SOM mRNA and IR in our SRS prone mice (42 days after AAV-*TeLC* injection). In spite of the strong increases in SOM mRNA and IR previously observed in KA treated rats and after kindling (Sperk et al., 1992; Schwarzer et al., 1996), we detected no change in SOM mRNA expression in the hippocampus (Supplementary Image 3). There was also no correlation between SOM mRNA levels and the number of SRS experienced by the mice. Similarly, SRS did also not induce an increase in SOM-IR in the molecular layer of the subiculum (Figure 8).

#### Cannabinoid receptor CB1

**FIGURE 9:**
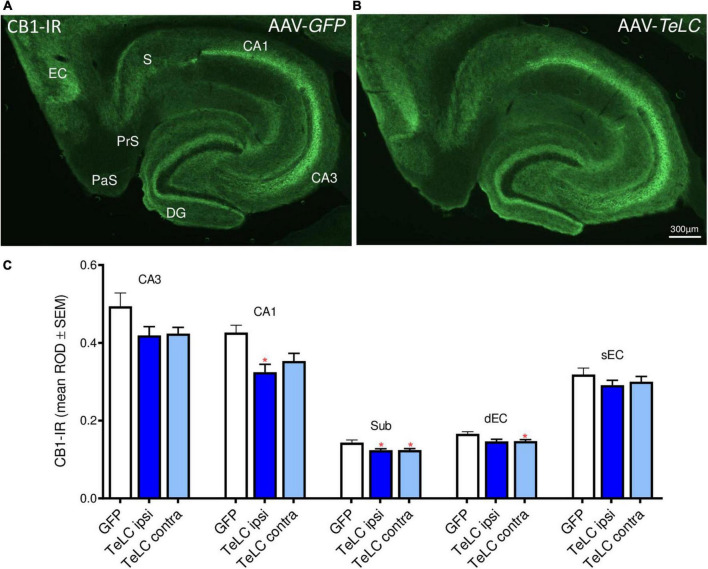
CB1-IR after AAV-*TeLC* injection. Immunofluorescence of horizontal sections on the level of the ventral hippocampus of mice injected into the subiculum with **(A)** AAV-*GFP* or **(B)** AAV-*TeLC*. CB1-IR is highly expressed in pyramidal layers of CA3, CA1, the subiculum and in the inner molecular layer of the dentate gyrus, layers that are innervated by basket cells. CB1-IR is presumably located presynaptically on these GABA-ergic terminals mediating inhibition of GABA release. **(C)** Optic density of CB1-IR is modestly reduced in sectors CA3 (statistically not significant) and CA1 (*p* < 0.05, ipsilateral side), in the subiculum (*p* < 0.05, *) and in the deep layers of the entorhinal cortex (*p* < 0.05, contralateral side). Note that the ROD values were not significantly different in the AAV-*TeLC*-injected side versus the not-injected contralateral area. Abbreviations in panels **(A,C)**: DG, dentate gyrus; EC, entorhinal cortex; dEC, deep entorhinal cortex; sEC, superficial entorhinal cortex; S, Sub, subiculum; PaS, parasubiculum; PrS, presubiculum.

CB1-IR was highly expressed in the pyramidal layers of CA1, CA3 and the subiculum, and in the inner molecular layer of the dentate gyrus. These are terminal areas of CCK-8 (and vesicular glutamate transporter, vGLUT3) positive basket cells containing the presynaptic CB1 receptors. As shown in Figure 9, we observed modest decreases in CB1-IR throughout the ventral hippocampal formation of mice that had experienced SRS during the 42 days observation period (see Figure 1). Since this decrease affects different parts of the hippocampus and is also present on the contralateral side it may be related to the SRS experienced and not directly to the unilateral vector injection.

## Discussion

### Plasticity of the NPY system

In the brain, the 36 amino acid-containing neuropeptide NPY is contained in large dense core vesicles of GABA-ergic interneurons. Whereas the classical transmitter GABA is already released after low frequency stimulation, neuropeptides like NPY are generally released only after sustained, high frequency stimulation (Hökfelt, 1991). In animal models of epilepsy and in human TLE, recurrent seizures cause marked overexpression of NPY in GABA-ergic interneurons and in glutamatergic mossy fibers (Sperk et al., 1992; Gruber et al., 1994; Mathern et al., 1995; Furtinger et al., 2001; Drexel et al., 2012). Together with the augmented expression of the GABA synthesizing enzyme GAD-1, overexpression of NPY indicates that the activity of the respective neurons persists beyond the acute seizures. It has also been suggested that high frequency stimulation during subsequent seizures may lead to enhanced release of NPY together with that of GABA (Gruber et al., 1994; Vezzani and Sperk, 2004). NPY is mostly released to extra-synaptic sites and inhibits glutamate release also through distant presynaptic Y2 receptors (Colmers et al., 1991; Greber et al., 1994). We therefore speculated that over-expressed NPY may serve as an endogenous anticonvulsive and protective mechanism (Marksteiner and Sperk, 1988; Vezzani and Sperk, 2004). Upon sustained neuronal stimulation during acute seizures, release of NPY may occur, so to say “on demand” and may dampen glutamate release and contribute to termination of seizures.

Here, we studied NPY expression in an animal model with rare but recurrent seizures (SRS) induced by selective silencing of GABA/PV containing interneurons in sector CA1 and subiculum (Drexel et al., 2017). This animal model is based on unilateral injection of small amounts of an AAV vector into transgenic PV-cre mice, resulting in local expression of tetanus toxin light chain only in PV containing cells. In the hippocampus and the subiculum, PV-containing basket cells and axo-axonic cells exert potent somatic and perisomatic inhibition of pyramidal cells. The toxin locally inhibits vesicular GABA release from the affected interneurons. Consequently, the mice develop SRS and IS clusters but show no neurodegeneration (Drexel et al., 2017). It is also important to note that only SRS but not recurrent clusters of IS caused increased NPY expression. Whereas expression of the peptide was transient in mossy fibers, it persisted in interneurons. This is consistent with our previous finding showing that after KA-induced seizures over-expression of NPY lasted for a prolonged time in interneurons, whereas it was limited to a few days in mossy fibers (Gruber et al., 1994). Interestingly, in our present experiments, the increase in NPY-IR in subicular interneurons (not in mossy fibers) persisted, although the frequency of SRS appeared to decline 4–6 weeks after initial silencing of GABA/PV neurons.

### Evidence for an endogenous protective effect of NPY

To demonstrate that endogenous over-expressed NPY serves a protective role, we injected the Y2 receptor antagonist JNJ 5207787 to our seizure prone mice. The mice had been injected with the AAV-*TeLC* into the sector CA1/subiculum before and SRS were triggered additionally by a low dose of PTZ after 7 days. After this treatment, all mice experienced SRS leading to upregulation of NPY (Drexel et al., 2017). Notably, these mice did not respond to injection of JNJ 5207787 alone, indicating that Y2 receptors were not occupied by NPY (NPY is almost not released prior to seizures). Thus, to induce NPY release in the mice, we challenged them by injecting once more a low dose of PTZ. Since the AAV-*TeLC*-injected mice (due to silencing of PV neurons) have a lowered seizure threshold (Drexel et al., 2017), the otherwise not convulsive dose of PTZ induced modest convulsions (Figure 7A). When applying the Y2 receptor antagonist in addition, we observed significant increases in PTZ-induced epileptic activity (Figures 7A,B). It is important again to point out that JNJ 5207787 *per se* did not evoke seizures, although the AAV-*TeLC*-injected mice had a lowered seizure threshold. Only triggering convulsions and thereby also the release of NPY disclosed the activity of the Y2 antagonist. This finding clearly suggests that over-expressed NPY is released during seizures and then exerts its anticonvulsive activity (antagonized by JNJ 5207787).

### Seizures induce release of NPY

The fact that seizures can induce NPY release has been shown using another experimental approach long ago. Measuring NPY levels in brain areas after a KA-induced status epilepticus revealed an initial drop in the levels of the peptide due to its extensive release and degradation during and after the acute seizures (Bellmann et al., 1991). This initial drop in NPY levels is overcome after 1–3 days and is followed by re-synthesis and marked over-expression of the neuropeptide (Marksteiner et al., 1990; Bellmann et al., 1991). Upregulation of the NPY, however, seems to require recurrent seizures or kindling (Schwarzer et al., 1996). Early anticonvulsant treatment with MK-801 or thiopental after the initial KA-induced status epilepticus prevented the subsequent increases in NPY and somatostatin levels (Marksteiner et al., 1990; Vezzani and Sperk, 2004).

### Earlier evidence for epileptic tolerance mediated by NPY expression

An association between epileptic tolerance and NPY upregulation has also been reported by El Bahh et al. (2001, 2005). They observed that intrahippocampal injection of a low dose of KA can prevent pyramidal cell loss that had been induced by subsequent intraventricular injection of the toxin, and that this effect correlated with increased NPY-IR in CA1 interneurons. Interestingly, this “preconditioning” lasted for seven but not for 15 days, again supporting the idea that NPY-induced epileptic tolerance may be limited in time and may require repeated convulsions for its persistence. Previous experiments demonstrating that febrile seizures induce tolerance to subsequent thermal induction of seizures indicate fast development of epileptic tolerance (Dubé et al., 2005). Also in these experiments, the inhibition of epileptic tolerance by application of a Y2 receptor antagonist suggested a crucial role of NPY over-expressed by the preceding seizures (Dubé et al., 2005).

### The role of interneurons vs. granule cells/mossy fibers

Over-expression of NPY in interneurons of the subiculum (possibly also of the cortex and dentate hilus) may lead to a lasting seizure protection. On the other hand, both NPY and Y2 receptors are also ectopically expressed in granule cells/mossy fibers after severe seizures (Gruber et al., 1994; Röder et al., 1996; Sadamatsu et al., 1998). Expression of the peptide as well of its Y2 receptors, however, is short-lived and wanes after a few days. It may, however, represent an immediate anticonvulsive mechanism protecting from acute epileptic activity that often persists even for several days after the initial status epilepticus. The underlying mechanism for NPY’s anticonvulsive activity is a potent inhibition of glutamate release from mossy fibers mediated by presynaptic NPY-Y2 receptors (Colmers et al., 1991; Greber et al., 1994). Interestingly, the anticonvulsive action of brain derived neurotrophic factor (BDNF) has been suggested to be mediated by inducing NPY expression (Reibel et al., 2000). The mechanisms underlying the different durations of expression in interneurons vs. granule cells are not known.

### Expression of VGAT, SOM, dynorphin and CB1 receptor after silencing PV neurons

In addition to NPY-IR, we investigated the expression of VGAT, SOM, dynorphin and CB1 receptor in the subiculum and hippocampus of mice that had experienced SRS and IS during a period of 42 days (Experiment 1). VGAT-, SOM- and dynorphin-IR and SOM mRNA were not altered indicating that there is no sprouting of the respective GABA neurons and of mossy fibers. In contrast, we observed a modest decrease of the endocannabinoid receptor CB1. This change could be associated with reduced inhibition of GABA release from basket cells and, thus, could also represent an endogenous anticonvulsive mechanism. In a preliminary experiment we also applied the CB1 agonist WIN55,212 to AAV-*TeLC*-injected mice. If the reduction in CB1 receptors would have an anticonvulsive effect, the CB1 agonist should be proconvulsive (in the same way as the Y2 antagonist). We, however, observed no effect of WIN55,212.

A key finding of the model of KA-induced recurrent seizures was the selective degeneration of PV-containing basket cells in the subiculum (Drexel et al., 2011) that may be causatively related to the subsequent development of SRS. This finding is supported by the present model showing that already unilateral silencing of PV-interneurons in the subiculum (without degeneration of the affected neurons) leads to SRS (Drexel et al., 2017). In the KA model, fibers of SOM neurons sprout in the subiculum [and even beyond to the molecular layer of the dentate gyrus (Peng et al., 2013)] whereas they do not in our present experiments after only silencing PV basket cells. SOM is primarily contained in oriens-lacunosum moleculare (O-LM) cells projecting from the stratum oriens to the stratum lacunosum moleculare in CA1 (and from the pyramidal cell layer to the molecular layer of the subiculum) and mediate feed-back inhibition upon pyramidal cells (Klausberger, 2009). NPY is mainly contained in bistratified cells and in Ivy cells also exerting feed-back inhibition upon pyramidal cell dendrites (Klausberger, 2009). It is interesting to note that NPY-containing axon terminals over-express the peptide (or even sprout) already after silencing of PV neurons (without neurodegeneration) and resulting SRSs, whereas SOM-containing O-LM cells only sprout after degeneration of PV neurons in the subiculum (KA model). Sprouted O-LM cells may mediate augmented feed-back inhibition after the loss of PV cell mediated feed-forward inhibition. As discussed above, in the present model over-expressed NPY may primarily act by presynaptic inhibition of glutamate release.

### Does NPY-induced epileptic tolerance also play a role in human TLE?

In specimens of TLE patients, less prominent expression of NPY was seen in granule cells/mossy fibers, possibly due to a prolonged interval between the last seizure and surgery in the patients (Furtinger et al., 2001). Expression of Y2 receptors—in contrast to Y1 receptors—however, was increased throughout the hippocampus including mossy fibers, and increased expression of NPY was observed in axons of interneurons of the subiculum and other parts of the hippocampus (Furtinger et al., 2001). In hippocampal slices obtained from TLE patients, NPY prominently inhibits glutamate release (Ledri et al., 2015). Thus, also in humans NPY may mediate epileptic tolerance, and enhancing NPY-Y2 receptor mediated transmission may be an effective strategy for antiepileptic treatment for epilepsies in humans (Cattaneo et al., 2020).

### Conclusion

In conclusion, we provided evidence that NPY over-expressed by SRS and released “on demand” by acute seizures may contribute to epileptic tolerance by stimulating Y2 receptors and inhibiting glutamate release. On the other hand, also increased release of GABA and NO from the same neurons could take part in this mechanism. The site of the anticonvulsive activity of endogenous NPY, however, may not only be limited to the hippocampus and subiculum since SRS cause lasting overexpression of NPY also in other cortical areas such as the perirhinal and entorhinal cortex (Drexel et al., 2012).

## Data availability statement

The raw data supporting the conclusions of this article will be made available by the authors, without undue reservation.

## Ethics statement

This animal study was reviewed and approved by Committee for Animal Protection of the Austrian Ministry of Science.

## Author contributions

MD performed the experiments in part assisted by technical personnel. GS planned the study. GS and MD prepared the manuscript. Both authors contributed to the article and approved the submitted version.

## Footnotes

1
http://imagej.nih.gov/ij

## Supplementary material

Supplementary Image 1 Dynorphin-IR is not altered in mossy fibers. Dynorphin-IR was assessed in the terminal field of mossy fibers in the stratum lucidum 42 days after AAV-*GFP* **(A)** or AAV-*TeLC* injection **(B)**. **(C)** Dentate hilus of the same section as in panel **(B)**; note the absence of mossy fiber sprouting proving that there is no neurodegeneration. **(D)** Relative optic densities (ROD) in the strata lucidum of AAV-*GFP*-injected controls (GFP) and of AAV-*TeLC*-injected mice without [TeLC (-sz)] and with previous SRS [TeLC (+sz)].

Supplementary Image 2 VGAT-IR in mice with previous SRS. VGAT immunofluorescence in horizontal sections on the level of the ventral hippocampus of mice injected with AAV-*GFP* **(A)** or AAV-*TeLC* **(B)**. **(C)** Mean VGAT-immunofluorescence levels were not altered in the outer molecular layer of the subiculum (OML) 42 days after AAV-*TeLC* injection and did not correlate with the number of previous seizures **(D)**. DG, dentate gyrus; EC, entorhinal cortex; PaS, parasubiculum; PrS, presubiculum; S, subiculum; OML, outer molecular layer.

Supplementary Image 3 SOM mRNA in mice with SRS. Horizontal sections on the level of the ventral hippocampus of mice injected with AAV-*GFP* **(A,C)** or AAV-*TeLC* **(B,D)** were subjected to *in situ* hybridization of SOM mRNA. **(E)** Mean SOM mRNA levels were not altered in interneurons located in the hilus of the dentate gyrus 42 days after AAV-*TeLC* injection. **(F)** SOM mRNA expression also did not correlate with the numbers of previous SRS.
