# Supplementary figures and images for: Seizure-induced overexpression of NPY induces epileptic tolerance in a mouse model of spontaneous recurrent seizures
Source: Front Mol Neurosci. 2022 Oct 13;15:974784. doi: 10.3389/fnmol.2022.974784 (PMC9608171; doi:10.3389/fnmol.2022.974784)

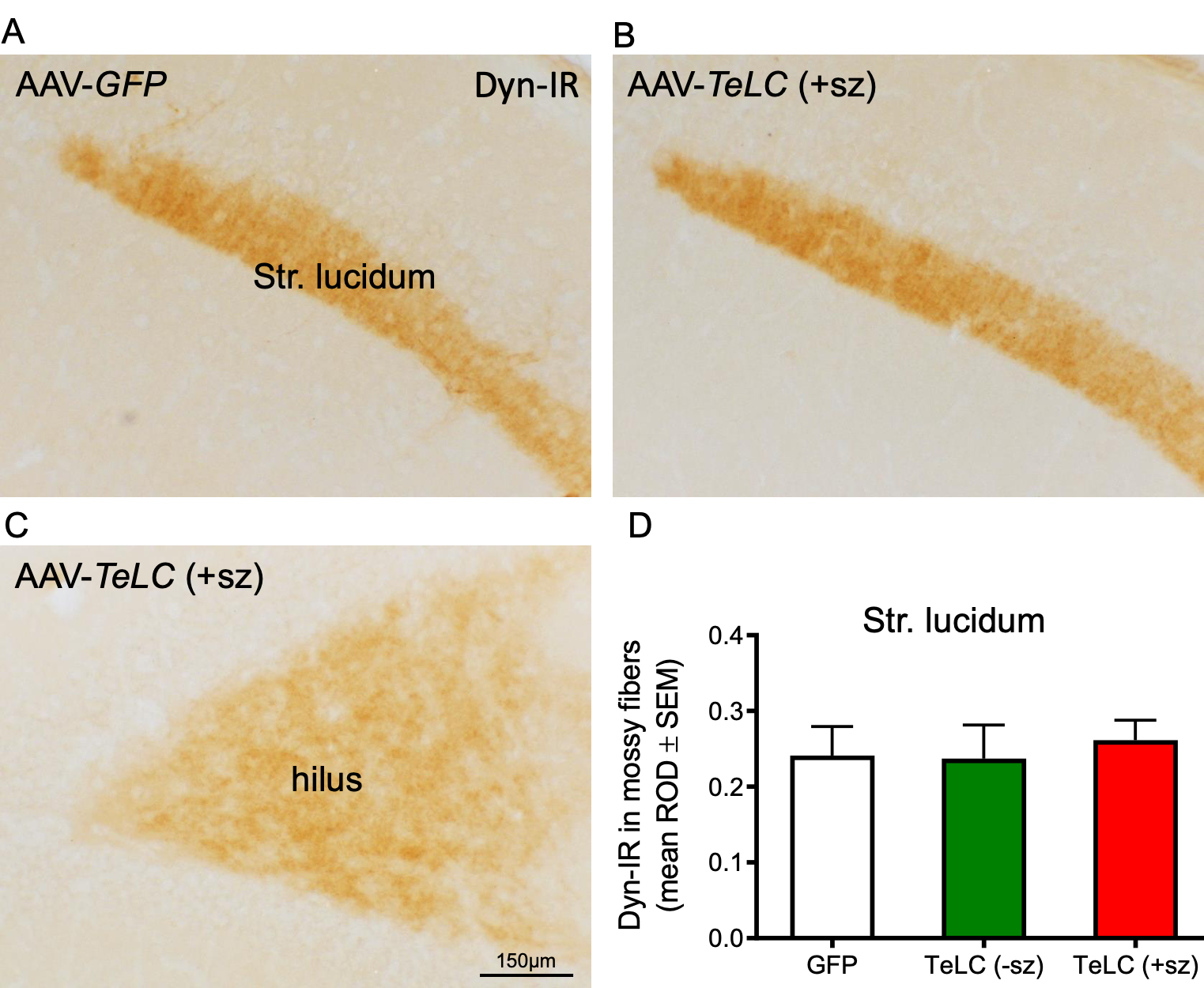

Supplement: Supplementary Image 1 — Dynorphin-IR is not altered in mossy fibers. Dynorphin-IR was assessed in the terminal field of mossy fibers in the stratum lucidum 42 days after AAV-GFP (A) or AAV-TeLC injection (B). (C) Dentate hilus of the same section as in panel (B); note the absence of mossy fiber sprouting proving that there is no neurodegeneration. (D) Relative optic densities (ROD) in the strata lucidum of AAV-GFP-injected controls (GFP) and of AAV-TeLC-injected mice without [TeLC (-sz)] and with previous SRS [TeLC (+sz)]. [file Image_1.tiff]

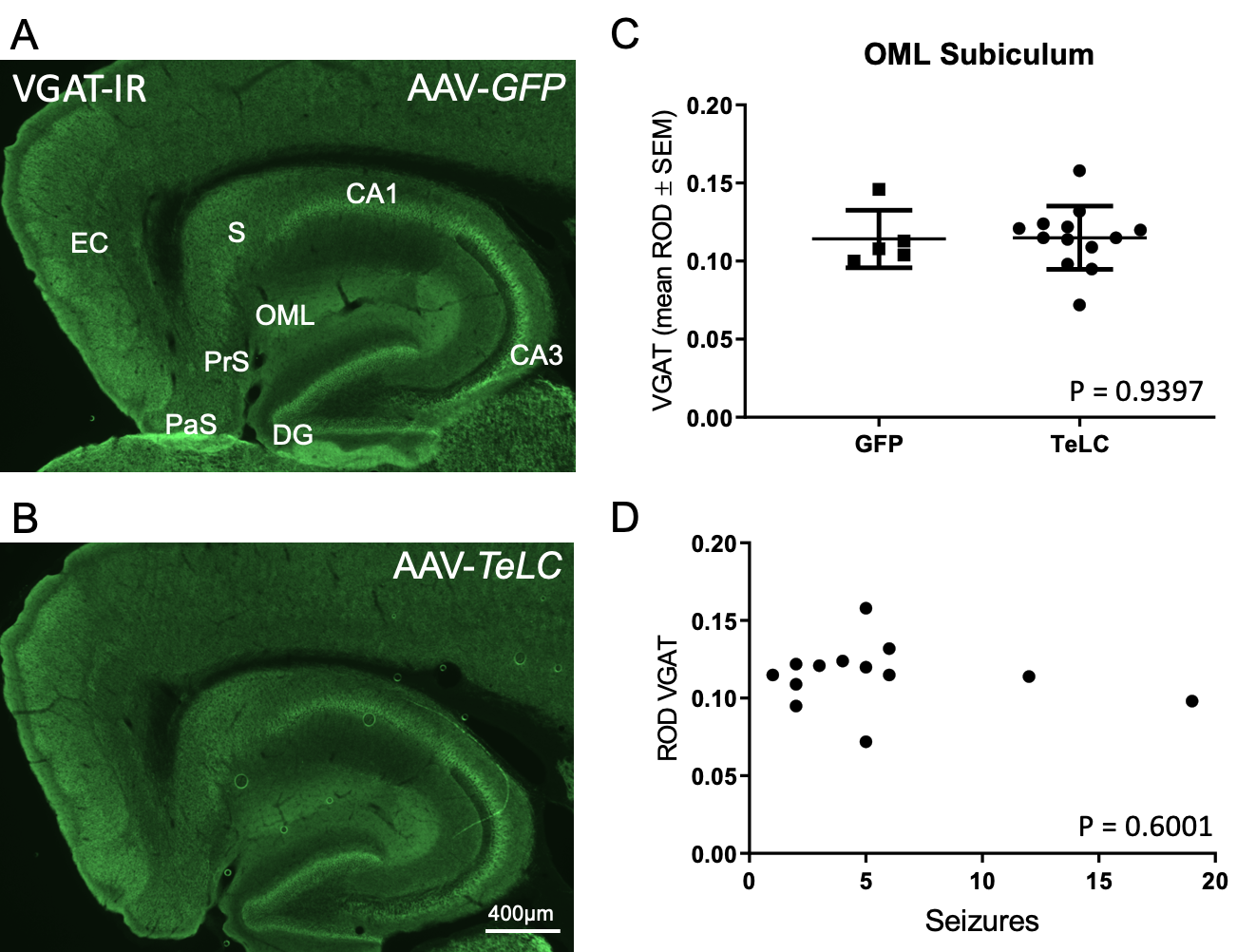

Supplement: Supplementary Image 2 — VGAT-IR in mice with previous SRS. VGAT immunofluorescence in horizontal sections on the level of the ventral hippocampus of mice injected with AAV-GFP (A) or AAV-TeLC (B). (C) Mean VGAT-immunofluorescence levels were not altered in the outer molecular layer of the subiculum (OML) 42 days after AAV-TeLC injection and did not correlate with the number of previous seizures (D). DG, dentate gyrus; EC, entorhinal cortex; PaS, parasubiculum; PrS, presubiculum; S, subiculum; OML, outer molecular layer. [file Image_2.tiff]

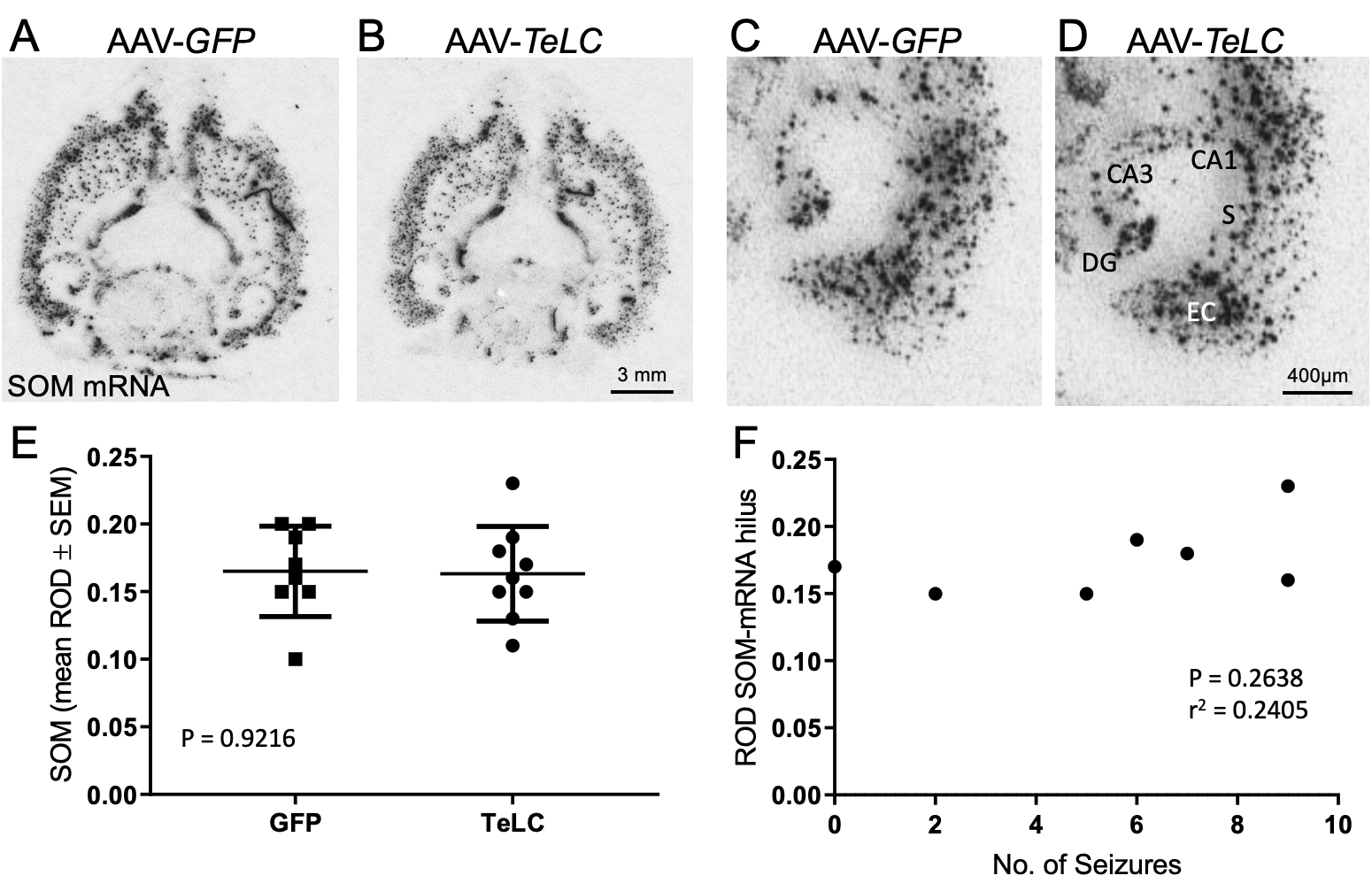

Supplement: Supplementary Image 3 — SOM mRNA in mice with SRS. Horizontal sections on the level of the ventral hippocampus of mice injected with AAV-GFP (A,C) or AAV-TeLC (B,D) were subjected to in situ hybridization of SOM mRNA. (E) Mean SOM mRNA levels were not altered in interneurons located in the hilus of the dentate gyrus 42 days after AAV-TeLC injection. (F) SOM mRNA expression also did not correlate with the numbers of previous SRS. [file Image_3.TIFF]
